# Gene and Its Promoter Cloning, and Functional Validation of *JmSOC1* Revealed Its Role in Promoting Early Flowering and the Interaction with the JmSVP Protein

**DOI:** 10.3390/ijms252312932

**Published:** 2024-12-01

**Authors:** Tianyi Dong, Mengmeng Zhang, Jingwen Wu, Jingze Li, Chunping Liu, Lijie Zhang

**Affiliations:** 1 College of Forestry, Shenyang Agricultural University, Shenyang 110866, China; 2 Key Laboratory of Forest Tree Genetics, Breeding and Cultivation of Liaoning Province, Shenyang 110866, China; 3 Key Laboratory of Silviculture of Liaoning Province, Shenyang 110866, China

**Keywords:** *JmSOC1*, heterodichogamous species, flower bud development, genetic transformation, promoter, genetic transformation, *Juglans mandshurica*, yeast one hybrid

## Abstract

*Juglans mandshurica*, a notable woody oil tree species, possesses both fruit and timber value. However, the complete heterodichogamous flowering mechanism in this species remains elusive. *SUPPRESSOR OF OVEREXPRESSION OF CONSTANS1 (SOC1)* is a crucial regulator of flower bud development in *Arabidopsis thaliana*. In this study, we cloned the coding DNA sequence (CDS) of the *JmSOC1* gene, revealing a 705 base pair (bp) sequence that encodes a protein of 234 amino acids. The JmSOC1 protein contains a highly conserved MADS-box domain, indicating its role as a transcription factor, and is predominantly localized in the nucleus. The *JmSOC1* gene expressed the highest in flower buds. The peak expression level of *JmSOC1* during the physiological differentiation phase occurred earlier in male flower buds of protandry (MPD) on April 10th compared to female flower buds of protandry (FPD) on April 14th; similarly, the peak expression in female flower buds of protogyny (FPG) on April 2nd preceded that in male flower buds of protogyny (MPG) on April 14th. This may be the primary reason for the earlier differentiation of the male flowers in protandry individuals and the female flowers in protogyny individuals. Overexpression of *JmSOC1* in wild-type *A*. *thaliana* resulted in earlier flowering, accompanied by an upregulation of key flowering-related genes such as *LEAFY* (*LFY*), *APETALA1* (*AP1*), and *FLOWERING LOCUS T* (*FT*). To further explore the function of *JmSOC1*, a 782 bp promoter sequence of *JmSOC1* gene was cloned, which has been verified to have promoter activity by GUS staining. Furthermore, the interaction between the *JmSOC1* gene promoter and its upstream regulatory protein JmSVP was verified using a yeast one-hybrid. These results offer valuable insights into the molecular mechanisms underpinning the promotion of early flowering in *J. mandshurica* and hold promise for laying a theoretical foundation for the flowering regulation network of this species.

## 1. Introduction

*Juglans mandshurica* is one of the ‘three major hardwood’ tree species in Northeast China [1], which holds vast development prospects due to its significant economic, nutritional, medicinal, and ornamental values. However, in recent years, due to human interference, it has been listed as a nationally protected rare and endangered tree species of Grade II in China. Prior research by our research team on the reproductive biology of *J*. *mandshurica* revealed that the species is a typical monoecious plant with heterodichogamous characteristics [2]. The alternating flowering and pollination of protandry (male flowers blooming first) and protogyny (female flowers blooming first) flowers in *J*. *mandshurica* effectively avoids self-pollination, which ensures genetic diversity and successful reproduction in the species.

The transition from vegetative growth to reproductive growth in trees is a crucial developmental process in the plant life cycle, which is essential for reproductive success. In the model plant *Arabidopsis thaliana*, five regulatory flowering pathways have been identified: vernalization, photoperiod, autonomous, gibberellin, and age pathways [3]. These pathways converge to regulate the overexpression of flowering locus genes *FLOWERING LOCUS T* (*FT*), *CONSTANS* (*CO*), and *FLOWERING LOCUS C* (*FLC*), as well as the expression of several flowering pathway integrator genes such as *SUPPRESSOR OF OVEREXPRESSION OF CO1* (*SOC1*) and *LEAFY* (*LFY*) [4,5].

*SOC1* integrates various flowering signals related to photoperiod, temperature, hormones, and age pathways [6,7]. In the vernalization pathway, the FLC protein forms a dimer with the flowering repressor SVP, which binds to the regulatory elements in the *SOC1* and *FT* promoters, inhibiting the expression of the *SOC1* directly or indirectly [8]. In the photoperiod pathway, the *CO* gene upregulates *SOC1* expression under long-day (LD) conditions to promote flowering [9]. Under short-day (SD) conditions, *SOC1* gene is positively regulated by gibberellins (GA) [10]. In the age-related pathway, miRNA156-targeted SQUAMOSA PROMOTER BINDING protein-like (SPLs) and other transcription factors such as *FT* and *AGAMOUS-LIKE24* (*AGL24*) contribute to the upregulation of *SOC1* gene [11,12,13].

*SOC1* encodes a MADS-box protein, which belongs to the *SOC1/TOMATO MADS3 (TM3)* clade of *MADS-box* genes, and its homologs have been described in multiple species [14,15,16]. *SOC1* gene is widely expressed in various tissues, including roots, leaves, shoot meristems, and floral organ primordia, and its expression increases during the flower development period [17]. Research has shown that overexpression of the *SOC1* gene leads to earlier flowering in some plant species such as in *Eucalyptus grandis* [18] and *Vaccinium corymbosum* [19].

To investigate the molecular regulatory mechanism of flower development in *J. mandshurica*, the coding DNA sequence (CDS) of *JmSOC1* was cloned, and the expression pattern of the *JmSOC1* during flower development was analyzed. To explore the function of the *JmSOC1*, an overexpression vector of *JmSOC1* was constructed and transformed into *A. thaliana*. Furthermore, the promoter sequence of *JmSOC1* gene was cloned, and the interaction between the promoter and its upstream protein JmSVP was verified using a yeast one-hybrid.

This study aims to elucidate the regulatory function of *JmSOC1* in the development process of *J*. *mandshurica* flower buds and the molecular mechanism that promotes early flowering. The results are expected to provide a theoretical foundation for the flowering regulation network and breeding of superior varieties of *J. mandshurica*.

## 2. Results

### 2.1. JmSOC1 Cloning and Sequence Analysis

The CDS of the *JmSOC1* gene spans 705 bp (accession number: PP601112), which encodes 234 amino acids (Table S1). The Blast alignment of the NCBI database shows that over 70% similarity between the *JmSOC1* and its homologous genes in other species. Furthermore, multiple sequence alignment revealed that the JmSOC1 protein belongs to the MADS-box and K-box families (Figure S1), with a highly conserved specific SOC1 motif containing 11 amino acid residues at the C terminus. The predicted nuclear localization signal motifs were also detected in the JmSOC1 amino acid sequence.

**Figure 1:**
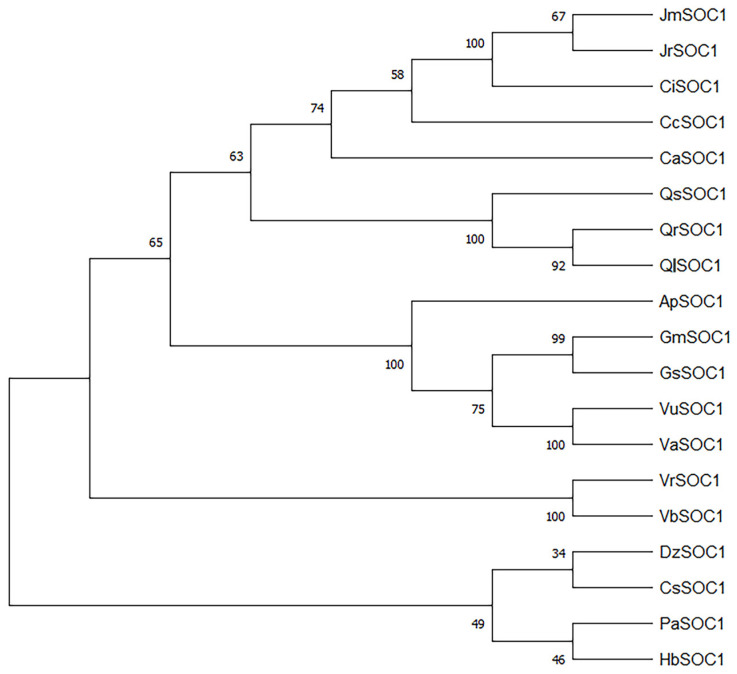
Phylogenetic analysis of amino acids sequences encoded by JmSOC1 and its homologous in other species. The phylogenetic tree for SOC1 was constructed using MEGA 7.0 software, using the Maximum Likelihood method with 1000 bootstraps. ApSOC1, *Abrus precatorius*, XP_027339460.1; CaSOC1, *Corylus avellana*, XP_059456278.1; CcSOC1, *Carya cathayensis*, AHI85950.1; CiSOC1, *Carya illinoinensis*, XP_042951334.1; CsSOC1, *Citrus sinensis*, XP_015389648.1; DzSOC1, *Durio zibethinus*, XP_022769695.1; GmSOC1, Glycine max, NP_001236377.1; GsSOC1, Glycine soja, XP_028247910.1, HbSOC1, *Hevea brasiliensis*, XP_021686250.2; JrSOC1, *Juglans regia*, XP_018820385.1; PaSOC1, *Populus alba*, XP_034904415.1; QlSOC1, *Quercus lobata*, XP_030930174.1; QrSOC1, *Quercus robur*, XP_050249824.1; QsSOC1, *Quercus suber*, XP_023900830.1; VaSOC1, *Vigna angularis*, XP_017417288.1; VbSOC1, *Vitis balansana*, WAX25730.1; VrSOC1, *Vitis riparia*, XP_034709248.1; VuSOC1, *Vigna umbellata*, XP_047167771.1.

The phylogenetic analysis revealed the evolutionary relationship among the JmSOC1 protein from different species. The JmSOC1 protein exhibits the closest with JrSOC1 from *J. regia*, subsequently followed by CiSOC1 from *Carya illinoinensis*, CcSOC1 from *Carya cathayensis*, CaSOC1 from *Corylus avellana*, QsSOC1 from *Quercus suber*, QrSOC1, *Quercus robur*, and QlSOC1 from *Quercus lobata* (Figure 1).

### 2.2. Subcellular Localization of JmSOC1

**Figure 2:**
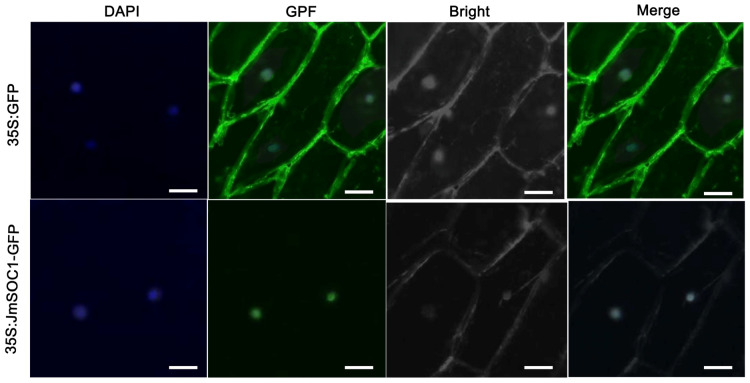
Subcellular localization of the JmSOC1-GFP fusion protein. The scale bars = 100 µm.

Subcellular localization of JmSOC1 was conducted via the bioistic PDS-1000/He Particle Delivery System, which transiently transformed onion epidermal cells with the pBI121-GFP vector and the fusion expression vector pBI121-*JmSOC1*-GFP. The control vector (pBI121-GFP) exhibited green fluorescence throughout the cell, while the recombinant vector pBI121-*JmSOC1*-GFP produced green fluorescence specifically within the nucleus (Figure 2), indicating that the JmSOC1 protein is localized in the nucleus.

### 2.3. JmSOC1 Expression Analysis

**Figure 3:**
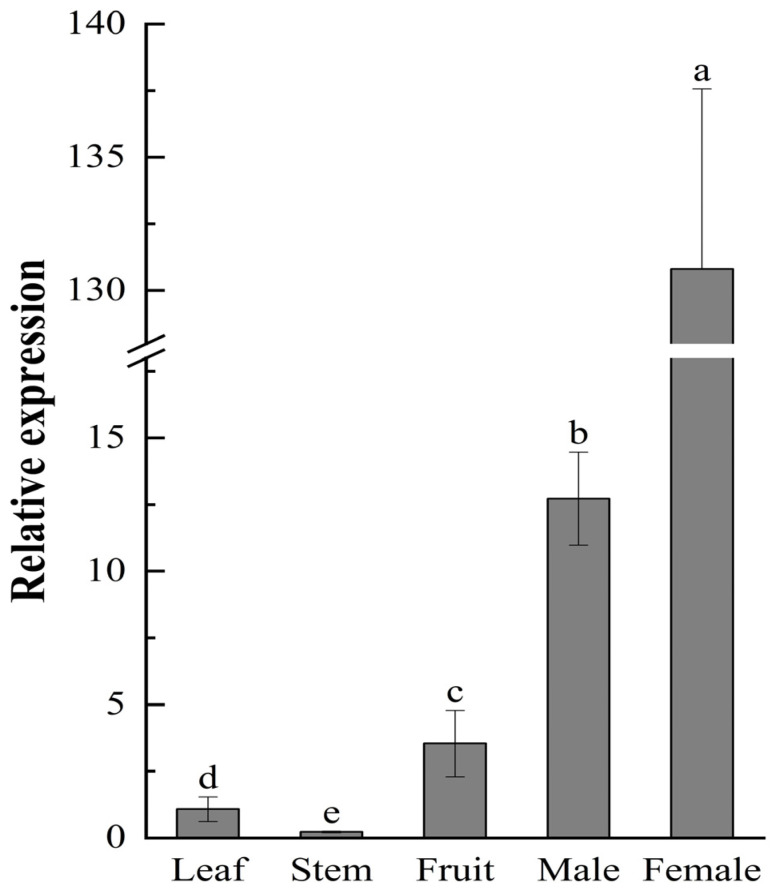
Expression pattern analysis of *JmSOC1* in different organs. The error bars represent the standard deviation of 3 replicates, the lowercase letters represent significant difference at α = 0.05 among different organs.

*JmSOC1* expressed in various organs and tissues; however, the expression level differed significantly (*p* < 0.05, Figure 3), which showed the highest expression in female flower buds, subsequently followed by male flower buds, fruits, leaves, and stems.

**Figure 4:**
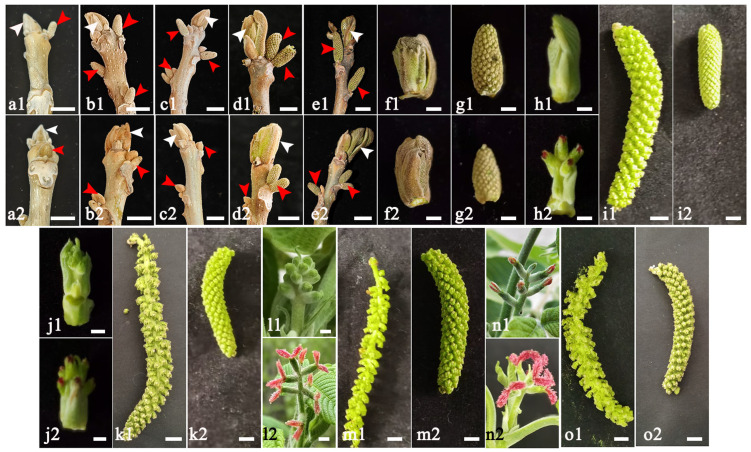
Morphologies of male and female flower buds of protandry and protogyny at different developmental stages. (**a1**–**e1**), The female and male flower bud of protandry on 2, 10, 14, 24 and 30 April 2022, respectively; (**a2**–**e2**), The female and male flower bud of protogyny on 2, 10, 14, 24 and 30 April 2022, respectively. (**f1**,**h1**,**j1**,**l1**,**n1**) represent the female flower bud of protandry on 4, 13, 17, 20 and 23 May 2022, respectively; (**g1**,**i1**,**k1**,**m1**,**o1**) represent the male flower bud of protandry on 4, 13, 17, 20 and 23 May 2022, respectively. (**f2**,**h2**,**j2**,**l2**,**n2**) represent the female flower bud of protandry on 4, 13, 17, 20 and 23 May 2022, respectively; (**g2**,**i2**,**k2**,**m2**,**o2**) represent the male flower bud of protogyny on 4, 13, 17, 20 and 23 May 2022, respectively. The white and red arrows in (**a**–**e**) represent the female and male flower bud, respectively. The scale bars = 1 cm.

**Figure 5:**
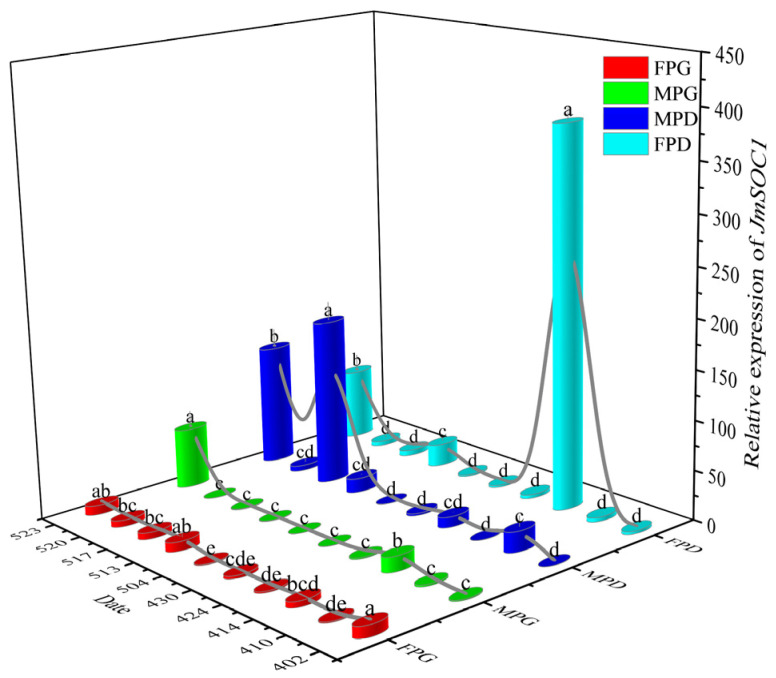
Expression patterns of *JmSOC1* in the physiological (2 to 30 April) and morphological (4 to 23 May 2022) differentiation stages of flower buds. FPG, female flower bud of protogyny; MPG, male flower bud of protogyny; MPD, male bud flower of protandry; FPD, female flower bud of protandry. The error bars represent the standard deviation of 3 replicates, lowercase letters represent significant difference at α = 0.05 among the different sampling stages of each flower bud.

The morphologies of male and female flower buds at different developmental stages of protogyny and protandry are shown in Figure 4. At the beginning of the physiological differentiation (2 April), the expression of *JmSOC1* genes varied significantly (*p* < 0.001, Figure 5) in the four types of flower buds, including the female flower bud of protogyny (FPG), the male flower bud of protogyny (MPG), the female flower bud of protandry (FPD), and the male flower bud of protandry (MPD). JmSOC1 gene expression was significantly higher in the two female buds (FPG > FPD, *p* < 0.05) than in the two male buds, while the expression differences in JmSOC1 were not significant in the two male flower buds (*p* > 0.05).

In both flower buds of protandry (MPD and FPD), the JmSOC1 expression showed three peaks. MPD peaked (20.70) earlier (10 April) than FPD (14 April, 381.47) during the physiological differentiation stage, but later than FPD (17 May, 166.91 vs. 13 May, 22.32) during the morphological differentiation stage. The third peak of both MPD (121.23) and FPD (71.01) occurred at the end of morphological differentiation (22 May).

In both flower buds of protogyny (MPG and FPG), the expression level of JmSOC1 in FPG reached its maximum (11.24) at the initial stage (2 April) of the physiological differentiation, and then underwent a process of sharp decline followed by a slow recovery, basically returning to its initial state in the middle and late stages of the morphological differentiation. In MPG, the expression level of JmSOC1 reached its first peak (15.10) in the middle stage of the physiological differentiation (14 April), followed by a sharp decline and stabilization, and then rose sharply again at the end of the physiological differentiation (22 May, 60.27), reaching its maximum value.

### 2.4. JmSOC1 Overexpression Promotes Flowering in Arabidopsis

**Figure 6:**
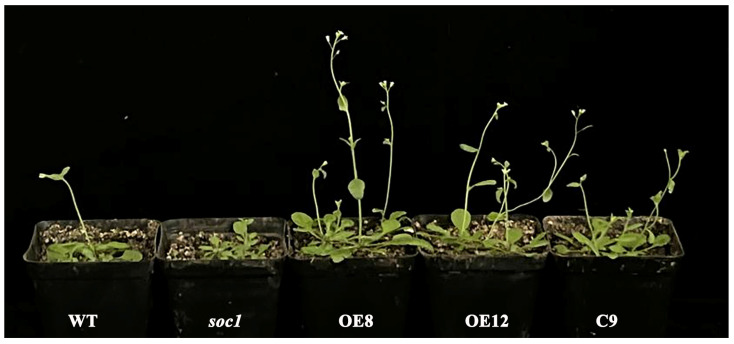
Phenotypic observation of *Arabidopsis thaliana* plantlets of wild-type (WT), *soc1* mutant (*soc1*), overexpressed *JmSOC1* plants (OE8 and OE12), and *soc1* mutant complemented with the *JmSOC1* (C9). Wild-type and *soc1* mutant seeds of *A*. *thaliana* (Columbia ecotype) were purchased from the TAIR website, all the plantlets used in this study are Columbia ecotype. The promoter used for overexpression and the *soc1* mutant is *CaMV 35S*.

Overexpression of *JmSOC1* in both wild-type (WT) *A. thaliana* and the *soc1* mutant resulted in distinct phenotypic changes, as shown in Figure 6. The statistics of bolting time, flowering time, rosette leaf number, secondary branches number, plant height, and the bolt diameter for T3 plants were summarized in Table 1. Compared to the WT, plants overexpressed with the *JmSOC1* gene (OE) exhibited earlier bolting and flowering times by approximately 5 d. Although the plants complemented with *JmSOC1* (C) flowered later than the WT, they still flowered significantly earlier than the *soc1* mutant by approximately 10 days.

The *soc1* mutant displayed an increase of approximately eight rosette leaves compared to the WT. In contrast, the C9 and OE12 plants showed a reduction in the number of rosette leaves compared to the *soc1* mutant and the WT, respectively. Similarly, the *soc1* mutant exhibited an increase in the number of lateral branches (10.67) compared to the WT (5.33). Also, the C9 and OE12 plants showed a reduction in number of the lateral branch compared to the *soc1* mutant and the WT, respectively, although this reduction was not significant in the OE12 plants.

The *soc1* mutant displayed a higher height (29.38 cm) compared to the WT (23.88 cm). Interestingly, the OE12 plants and the C9 plants showed a different performance, which showed an increase in height of OE12 (26.56 cm) compared to the WT, but decreased for C9 plants (25.37 cm) compared to *soc1* mutant (Table 1).

For the diameter of bolting, there is no significant difference between the WT and the *soc1* mutant; however, both the C and the OE plants showed an increase in diameter of bolting compared to the soc1 mutant (0.2 cm) and the WT (0.36 cm), respectively (Table 1).

**Figure 7:**
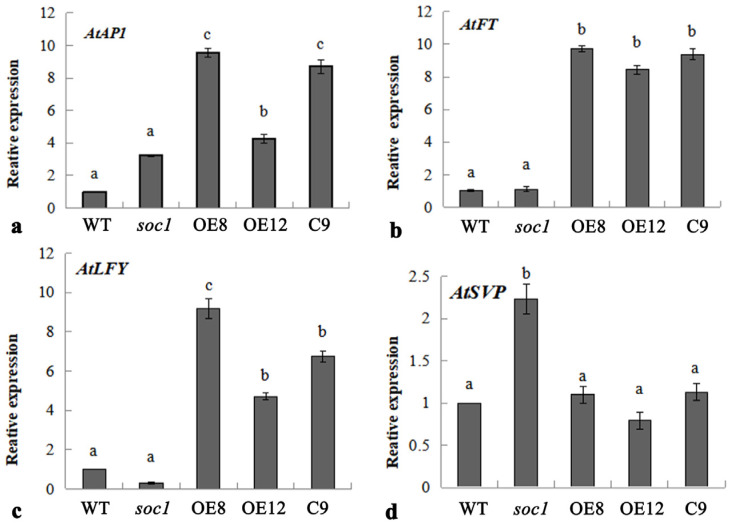
Expression levels of endogenous genes related to floral development in transgenic *Arabidopsis thaliana* plants expressing *JmSOC1*. a, *AtAP1*; b, *AtFT*; c, *AtLFY*; and d, *AtSVP*. WT, wild-type; *soc1*, *soc1* mutant; OE, overexpressed *JmSOC1* plants; C, *soc1* mutant complemented with the *JmSOC1*. The error bars represent the standard deviation of 3 replicates, lowercase letters represent significant difference at α = 0.05.

To gain insight into the molecular mechanism of *JmSOC1* regulating flowering time, the expression patterns of flowering regulatory genes *AP1*, *FT*, *LFY*, and *SVP* were analyzed in different sources of *Arabidopsis thaliana* under long-day (LD) conditions. The expression levels of *AtAP1*, *AtLFY*, and *AtFT* genes were not significantly different between the WT and *soc1* mutant, but were significantly higher in the OEs and C9 plants compared to the WT and soc1 mutant, respectively (Figure 7a–c). The expression level of the *AtSVP* gene in *soc1* mutant is significantly higher than that in WT, but this increased expression is attenuated in the C9; however, overexpressed the *JmSOC1* gene in the WT does not alter the expression of the endogenous *AtSVP* gene. (Figure 7d).

### 2.5. JmSOC1 Promoter Cloning and Bioinformatics Analysis

The promoter clone yielded a 998 bp sequence, which had an overlap of 216 bases with the 5′ end of the cDNA of *JmSOC1* gene. A total of 782 bp of the promoter sequence was obtained after removing the overlapped sequences. The promoter sequence of the *JmSOC1* gene shares over 90% similarity with the upstream sequence of *JrSOC1* homologs published on NCBI, with some deletions and mutations of bases (Figure S2).

Analysis of the cis-acting elements of the *JmSOC1* promoter reveals the presence of various cis-acting elements, including core cis-acting elements such as CAAT-box and TATAbox; flowering-related elements, including POLLEN1LELAT52, a cis-acting element specifically expressed in pollen; GTGANTG10, a promoter element for late pollen genes; and CArG-box motif, a binding site for flowering-related proteins; light-responsive elements, including Box4, G-Box, and GT1-motif; hormone-responsive elements, such as P-box and TATC-box; stress-responsive elements, such as LTR, a cis-acting element related to low-temperature responsiveness; MBS, a MYB binding site induced by drought; and ARE, an anaerobic induction regulatory element, and WRE3, a wound-responsive element (Table S2).

### 2.6. Analysis of the Promoter Activity of the JmSOC1 Promoter

**Figure 8:**
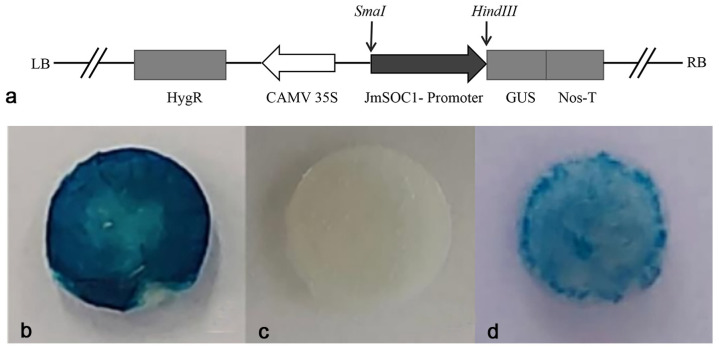
p1301-pJmSOC1 vector construction and GUS histochemical staining of transiently transformed tobacco leaves. (**a**) Schematic diagram of p1301-pJmSOC1 vector; (**b**) empty pCAMBIA1301 vector (positive control); (**c**) without pCAMBIA1301 vector (negative control); and (**d**) p1301-pJmSOC1.

The schematic diagram of the vector is shown in Figure 8a. *GUS* histochemical staining showed that the leaves of the positive control pCAMBIA1301 displayed significant blue staining (Figure 8b), while the leaves of the negative control remained unstained (Figure 8c). The leaves infected by p1301-pSOC1 turned blue (Figure 8d), but the color intensity was less than that of the positive control. This indicates that the JmSOC1 gene promoter possesses promoter activity.

### 2.7. Point-to-Point Validation by a Yeast One-Hybrid of JmSVP and JmSOC1 Promoter

**Figure 9:**
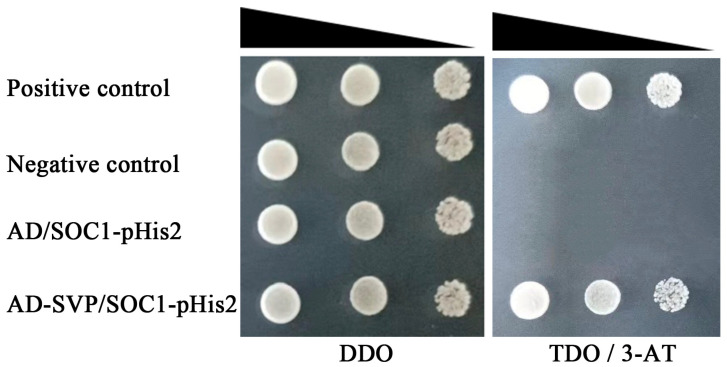
Point-to-point verification of a yeast one-hybrid for co-transforming yeast plasmid of JmSVP and *JmSOC1* promoter. DDO and TDO/3-AT means Synthetic Dropout Media (SD) with Double Dropout Supplements (DDO, SD/-Leu/-Trp), and Triple Dropout Supplements (TDO, SD/-His/-Leu/-Trp) with 3-AT. Positive control, pGADT7-rec2-p53 + pHIS2-p53; negative control, pGADT7-Rec2 + pHIS2-p53; self-activation assay, pGADT7-Rec2 + pHIS2-SOC1; interaction assay, pGADT7-SVP + pHIS2-SOC1.

Point-to-point validation by the yeast one-hybrid of JmSVP and *JmSOC1* promoter showed that all co-transformed yeast plasmid grew well on the DDO medium, which suggested that the co-transformations are successful (Figure 9). The strains of the negative control (pGADT7-Rec2 and pHIS2-p53) and the self-activation (pGADT7-Rec2 + pHIS2-SOC1) could not grow on TDO medium supplied 3-AT, and the positive control (pGADT7-rec2-p53 + pHIS2-p53) and the interaction assay (pGADT7-SVP + pHIS2-SOC1) grew well on TDO medium supplied 3-AT, which indicated the existence of interaction between JmSVP and *JmSOC1* promoter.

## 3. Discussion

The transition of higher plants from vegetative growth to reproductive growth marks the end of the juvenile phase of plants. Therefore, flowering is a crucial process in the life cycle of plants. Studies on *A*. *thaliana* have proven that the *SOC1* gene, as well as its homologous genes, which encodes MADS-box transcription factors, is mainly expressed in developing leaves and meristems, serving as an important integrator of flowering control in the vernalization pathway [20], photoperiod pathway [21], thermosensitive pathway [22], gibberellin pathway [23], autonomous pathway [24], and age pathway [25].

In this study, we cloned the *JmSOC1* gene and its promoter sequences. Subcellular localization analysis revealed that *JmSOC1* is primarily localized in the nucleus, which is consistent with studies conducted in other plants such as *Litchi chinensis* [26], *Ananas comosus* [27], and *Pyrus bretschneideri* cv. Dangshansuli [28]. Similarly to the promoter sequence of *AcSOC1* in *Ananas comosus*, the promoter sequences of *JmSOC1* contained core cis-acting elements such as CAAT-box and TATA-box, as well as flowering-related elements, CArG-box motif, light-responsive elements, hormone-responsive elements, stress-responsive elements, MBS, ARE and WRE3, a wound-responsive element. Based on this analysis, it can be inferred that the expression of the *JmSOC1* gene may be regulated by multiple factors.

Generally, *SOC1* gene is expressed in multiple organs or tissues such as roots, stems, leaves, flower buds, inflorescences, etc. The expression level of *SOC1* was usually the highest in mature leaves in *A. thaliana* [29], *Mangifera indica* [30], ‘Gannan’ naval orange [31], and in *Litchi chinensis* [26]. In *Ginkgo biloba*, a dioecious species, the expression level of *GbMADS6* (a *SOC1* homolog gene) in leaves than in female and male flowers [5]. However, in *Bambusa oldhamii*, the highest expression of *SOC1* was exhibited in flower buds and the lowest expression in stems [32]. These results indicate that *SOC1* is differentially expressed in various organs in different plant species. Similarly to that in *B. oldhamii*, in our study, the highest expression of *JmSOC1* was observed in female flower buds, subsequently followed by male flower buds, fruits, leaves, and stems. These results indicate that the *JmSOC1* participates in the process of flower bud development, especially in female flower bud development in *J*. *mandshurica*. The different expression patterns of *SOC1* in different organs may also be caused by the differences in sampling time. Studies have shown that with the prolongation of plant growth, *SOC1* is more likely to accumulate in leaves [30].

During the floral bud differentiation stage, the expression pattern of *SOC1* varied among different species. In *Citrus sinensis*, *CsSOC1* showed the highest expression level at 75 d before the full bloom stage. As the floral buds develop, the expression level exhibits a down-up-down-up trend. However, the expression levels at the later stages are significantly lower than that of the former [31]. During the floral bud differentiation of *Magnolia liliflora*, the expression patterns of the two *SOC1* genes exhibit a significantly lower level of expression during the middle to late stages (15th May–14th June) compared to the initial stages (5th April–10th May) [33]. In contrast, during the floral bud differentiation in *Ginkgo biloba*, the expression of the *SOC1* gene shows a gradual upward trend [34].

As *J*. *mandshurica* is a heterodichogamous species, we investigated the expression patterns of *JmSOC1* in four types of floral buds during different differentiation stages. In the two types of floral buds with protandry, the peak expression of *JmSOC1* during the physiological differentiation phase appeared earlier in MPD (10th April) compared to FPD (14th April). Similarly, in the two types of floral buds with protogyny, the peak expression of *JmSOC1* in FPG (2nd April) occurred earlier than in MPG (14th April). This may be the primary reason for the earlier differentiation of male flowers in protandry individuals and the earlier differentiation of female flowers in protogyny individuals. In *Cyclocarya paliurus*, another heterodichogamous species, genes that are related to plant hormones synthesis and response, glucose metabolism, as well as transcription factors, particularly those belonging to the MIKC family, play significant roles in regulating the asynchronous development of male and female flowers in the same mating type [35]. In *Ginkgo biloba* the expression level of *GbMADS6* in apex stems increased at initial differentiation stage of both female and male flower bud and decreased gradually along with flower bud development [5]. Nevertheless, to date, no research has been reported on the expression pattern of the *SOC1* gene in heterodichogamous plants.

Overexpression of *SOC1* gene have been confirmed to promote early flowering in many species, such as in *Vaccinium corymbosum* [36], *Medicago truncatula* ((Jaudal et al., 2018) [37], *Pyrus bretschneideri* [38], *Brassica rapa* [39] and *Litchi chinensis* [26]. In this study, *soc1* mutant *A. thaliana* showed delayed bolting and flowering than the WT plants, and the OE and C plants with *JmSOC1* exhibited earlier bolting and flowering compared to the WT and *soc1* mutant plants, respectively. These results revealed the roles of *JmSOC1* for earlier flowering.

Our research results show that the plant height of both the *soc1* mutant and the OE plants is higher than that of the WT, but the height of C plants is significantly lower than that of *soc1* mutant. No obvious difference in bolt diameter between the WT and the *soc1* mutant; however, the bolt diameter of OE and C plants increases significantly compared to the WT and the *soc1* mutant, respectively (Table 1). In *Medicago truncatula*, overexpression of *MtSOC1a* promoted main stem elongation, while the mutants exhibit shortened main stem [37]. In *Brassica campestris*, gibberellins (200 mg/L) and low temperature (4 °C, 15 °C) increased the plant height, and reduced the bolt diameter, which may be regulated by down-regulation of the expression levels of *BcSVP* and up-regulation of the expression levels of *BcSOC1-1*, *BcSOC1-2* [40]. These results indicate that the regulation of *SOC1* on plant height and bolt diameter are relatively complex.

Generally, in *A. thaliana*, the *FT* gene functions upstream of *SOC1* and positively regulates its expression [41]. Conversely, *AP1* [42] and *LFY* [43] functions downstream of *SOC1* and is positively regulated by *SOC1*. In this study, no significant difference in the expression of endogenous *AtFT*, *AtFT1*, and *AtLFY* were observed between the WT and the *soc1* mutant plants. This may be due to *soc1* mutation not affecting the regulatory region of these genes, or the changes caused by the mutation are compensated by other regulatory mechanisms. However, the OE and C plants with *JmSOC1* exhibited a significant increase in the expression of these genes. Based on our predictions, the expression products of *JmSOC1* gene may function as signaling molecules or regulatory factors, thereby influencing the expression of these genes.

In contrast, the higher expression level of *AtSVP*, a gene *negatively regulating flowering,* was detected in the soc1 mutant compared to the WT plant. The C plants complemented with *JmSOC1* reduced the expression of *AtSVP*; however, the overexpression of *JmSOC1* had no obvious effect on *AtSVP* expression. The results indicated that *JmSOC1* has a probably negative regulatory on *AtSVP*, and this negative regulatory relationship may exhibit a dosage effect. However, this result contradicts the known regulatory relationship between *SVP* and *SOC1*. Generally, in *A. thaliana*, *SVP* functions upstream of *SOC1*, which forms a complex with *FLOWERING LOCUS C* (*FLC*), binding to the CArG region of the *SOC1* promoter to inhibit its transcription, and then repressing plant flowering [44]. Our results about yeast one hybrid also confirmed the interaction of JmSVP and the promoter of *JmSOC1*. We speculate that this contradictory result may be due to the interactions between *SOC1* and *SVP*, or the existence of an unknown and complex regulatory network involving the two genes and other genes, proteins, or signaling molecules. However, to date, no relevant research has been able to provide a reasonable explanation for this phenomenon. This necessitates our continued efforts to conduct more in-depth research.

In *Arabidopsis*, the AtSVP protein binds to a specific CArG motif from the *AtSOC1* promoter, thereby inhibiting the expression of *AtSOC1*. The mutation of the specified CArG1 motif almost abolished the repression of *SOC1*. The *soc1* mutant of *Arabidopsis* exhibits a significantly delayed flowering time (28 leaves) compares to the wild type (13.2 leaves), while complementation with the *AtSOC1* gene, including approximately 2000 bp of the promoter sequence, partially restores the late-flowering phenotype to 15.4 leaves. However, when the specific CArG motif in the promoter sequence is mutated, the flowering time of the complemented *Arabidopsis* is earlier than any other plants (11.8 leaves). These results demonstrate that the AtSVP binding site at CArG motif is responsible for repressing *AtSOC1* during flowering [45]. In this research, our results indicate that in *J. mandshurica*, the JmSVP protein can also bind to *pJmSOC1*. However, further experiments are required to elucidate how the binding of JmSVP protein to *pJmSOC1* regulates *JmSOC1* expression and affects plant flowering.

## 4. Materials and Methods

### 4.1. Plant Materials

The plant materials were collected from a natural *J*. *mandshurica* forest located in the experimental forest farm of Liaoning Province in 2022. Samples of leaves, stem segments, and female and male flower buds were taken during the physiological differentiation stage (2, 10, 14, 24 and 30 April) and the morphological differentiation stage (4, 13, 17, 20 and 23 May). Additionally, mature fruits were sampled in early September. These samples were promptly frozen in liquid nitrogen and stored at −80 °C for future use.

Wild-type and *soc1* mutant seeds of *A*. *thaliana* (Columbia ecotype) were purchased from the TAIR website (https://www.arabidopsis.org/index.jsp (accessed on 8 March 2023)). The *soc1* mutants were verified using the triple primer method. The homozygosity of the mutant was verified using the three-primer method (Figure S3a), which showed a late-flowering phenotype (Figure S3b).

### 4.2. RNA Extraction and SOC1 Cloning

RNA was extracted from female and male flower buds using an RNAsecure Plant Kit (TIANGEN), and cDNA was synthesized subsequently according to the protocol of the StarScript III All-in-one RT Mix with gDNA Remove reverse transcription kit (GenStar). Forward (*SOC1*-F1) and reverse (*SOC1*-R1) primers (Table S3) were designed based on the *SOC1* sequence derived from the transcriptome of *J. mandshurica* flower buds. Subsequently, *SOC1* was cloned by PCR amplification using cDNA as a template. The PCR reaction mixture consisted of cDNA 1 uL, LA Taq Mix 12.5 µL, SOC1-F (10 µmol/L) 1 µL, SOC1-R (10 µmol/L) 1 µL, ddH_2_O 9.5 uL. The PCR reaction procedure is as follows: initial denaturation at 94 °C for 5 min, followed by 35 cycles of 94 °C for 30 s, 58 °C for 1 min, and 72 °C for 2 min, with a final extension at 72 °C for 7 min.

The PCR product was detected by 1% agarose gel electrophoresis and subsequently purified using the MiniBEST Agarose Gel DNA Extraction Kit Ver. 4.0 (TaKaRa). The purified PCR product was then ligated into the pMD-18-T Vector (Takara) according to the manufacturer’s instruction. The recombinant vector was then transformed into *E. coli* DH5α competent cells, and positive clones were selected on plates containing 50 mg/L Ampicillin (Amp). These clones were further subjected to PCR amplification and sequencing, and the correctly recombined vector confirmed by sequencing was designated as pMD18-T-SOC1.

### 4.3. Bioinformatics Analysis of JmSOC1

The obtained sequence was analyzed using NCBI Blast (http://blast.ncbi.nlm.nih.gov/Blast.cgi (accessed on 7 June 2022)), bioinformatics analysis was conducted using DNAMAN6.0, and phylogenetic tree for SOC1 was constructed using MEGA 7.0 software.

### 4.4. Subcellular Localization for JmSOC1

The primers used for the amplification of *JmSOC1* gene are designated as *SOC1*-F2 and *SOC1*-R2 (Table S3). The PCR product was ligated to the pBI121-GFP vector to generate the pBI121-*JmSOC1*-GFP. Both pBI121-GFP (serving as a positive control) and pBI121-*JmSOC1*-GFP were transiently transformed into onion (*Allium cepa*) epidermal cells using 5 μg of plasmid DNA via the bioistic PDS-1000/He Particle Delivery System (Bio-Rad, Hercules, CA, USA).

### 4.5. JmSOC1 Expression Patterns in J. mandshurica

Total RNA was extracted from the leaf, stem, and fruit at different developmental stages of female and male buds as described in Section 2.2. qRT-PCR primers (*DSOC1*-F and *DSOC1-R*) were designed based on the obtained *JmSOC1* gene sequence using Primer Premier 5.0 (Table S3). *Actin* was used as a reference gene and the corresponding primers are listed in Table S3. The expression level of *JmSOC1* in different organs was detected using the Applied Biosystems 7500 real-time PCR system (Applied Biosystems, Foster, CA, USA) with a UltraSYBR mixture (CWBIO).

The PCR procedure consisted of an initial denaturation step at 95 °C for 10 min followed by 40 cycles of 95 °C for 10 s and 60 °C for 30s. The fluorescence signals were collected in triplicate with 3 biological replicates, and the relative expression levels were calculated using the 2^−△△Ct^ method.

### 4.6. Expression Vector Construction and Transformation of JmSOC1 into A. thaliana

The cloned *JmSOC1* was amplified using primers *SOC1*-F3 and *SOC1*-R3 (Table S3) and the PCR products were ligated to the binary plant transformation vector pBI121. The corrected recombined vector was named pBI121-SOC1 and transformed into *Agrobacterium* GV3101, which was then transformed into WT and *soc1* mutants of *A. thaliana* by inflorescence infection method. The transformed plantlets of T1 and T2 generations were screened using Kanamycin (Kan) (Figure S4). The resistant plants of T1, T2, and T3 generations were confirmed by PCR (Figure S5). Phenotypic observations and gene expression analysis were performed for the wild-type, *soc1* mutants as well as the overexpressing clones OE12, and the complemented clone (C9) using T3 generations. Twelve plants were analyzed for each treatment.

### 4.7. Expression of Flowering-Related Genes in Transformed A. thaliana

RNA from WT, *soc1* mutants, and overexpression and complement plantlets of *A. thaliana* was extracted using TRIzol reagent (Invitrogen, Carlsbad, CA, USA). The expression level of flowering-related genes including *AP1* (Gene locus: AT1G69120), *FT* (Gene locus: AT1G65480), *LFY* (Gene locus: AT5G61850), and *SVP* (Gene locus: AT2G22540) were analyzed as described in Section 2.5, *Actin* (Gene locus: AT3G18780) was used as a reference gene. The primers used for qRT-PCR are listed in Table S3.

### 4.8. DNA Extraction and JmSOC1 Promoter Cloning

DNA was extracted from leaves of *J. mandshurica* using modified CTAB method. According to the CDS of the *JmSOC1* gene (accession number: PP601112), a comprehensive search was conducted in the *Juglans regia* database (NC_049909.1), revealing a 2000 bp non-coding nucleotide sequence located upstream of the SOC1 homologue. From this sequence, a specific 800 bp fragment containing the cis-acting element that binds to the SVP protein was selected. Specific primers *SOC1*-P-F1 and *SOC1*-P-R1 (Table S3) were designed to clone the *JmSOC1* promoter sequence. PCR amplification was conducted using DNA as a template with the reaction mixture of DNA 1 µL, LA Taq Mix 12.5 µL, SOC1-P-F1 (10 µmol/L) 1 µL, SOC1-P-R1 (10 µmol/L) 1 µL, and ddH_2_O 9.5 µL, and the reaction procedure followed the same protocol outlined in Section 2.2. Subsequent steps, including PCR product recovery, vector ligation, transformation, and positive clone screening, were also executed according to the procedures detailed in Section 2.2. The promoter sequence was aligned using DNAMAN 6.0, and then the regulatory elements within the promoter were predicted and analyzed using online tools such as Plant CARE (http://bioinformatics.psb.ugent.be/webtools/plantcare/html/ (accessed on 16 August 2022)) and PLACE (https://www.dna.affrc.go.jp/PLACE/ (accessed on 18 August 2022)).

### 4.9. JmSOC1 Promoter Vector Constructs and Activity Analysis

The *JmSOC1* promoter sequence was amplified using SOC1-P-F2 and SOC1-P-R2 (Table S3). The PCR products and the pCAMBIA1301 vector, were dual-digested with Hind III and Bgl II rapid endonuclease, and then ligated using T4 DNA Ligase. The recombined vector was then transformed into *E. coli* DH5α competent cells. Positive clones were selected using 50 mg/L Kan and subjected to PCR amplification for verification. The PCR products were subsequently sequenced to confirm the accurate insertion of the *JmSOC1* promoter into the pCAMBIA1301 vector. Upon successful sequencing confirmation, the correct recombined vector was designated as p1301-pSOC1.

The transformation of the recombinant vector p1301-pSOC1 into *Agrobacterium* EHA105 competent cells, as well as the subsequent preparation of the infection solution, was performed following the methodology outlined by Zhang et al. [43]. Additionally, the transient transformation of p1301-pSOC1 into *Nicotiana benthamiana* was also carried out according to the same protocol described in the reference.

### 4.10. Yeast One-Hybrid

The prey vector was constructed using yeast vector pGADT7-Rec2 and pMD19-T-JmSVP (conserved by Key Laboratory of Forest Tree Genetics and Breeding of Liaoning Province). The upstream and downstream primers ADSVP-F and ADSVP-R were designed based on the *JmSVP* sequence and SmaI site and its flanking sequences of pGADT7-Rec2 vector (Table S3). Plasmid DNA from pMD19-T-*JmSVP* was extracted and amplified by PCR using primers of ADSVP-F and ADSVP-R. Simultaneously, plasmid of pGADT7-Rec2 was extracted and digested using the restriction endonuclease SmaI, the products were recovered, purified, and then ligated to the PCR products of pMD19-T-Jm*SVP* target fragment using the NovoRec plus One step PCR Cloning Kit. The ligation products were transformed into *E. coli* Top10 competent cell, and the recombinant vectors were screened through PCR and sequencing to confirm their correctness. The verified recombinant vector was designated as the prey vector for yeast one-hybrid assays and named pGADT7-SVP.

The bait vector was constructed using *JmSOC1* promoter and the yeast vector pHIS2, the primers HISpSOC1-F and HISpSOC1-R were designed based on the *JmSOC1* promoter sequence and the EcoR I and Mlu I restriction sites of pHIS2 (Table S3). The *JmSOC1* promoter was amplified, recovered, and purified followed by double digestion with EcoRI and MluI restriction enzymes. Concurrently, the pHIS2 vector was also digested with EcoRI and MluI, generating a linear vector fragment. The digested JmSOC1 promoter fragment was then ligated to the linearized pHIS2 vector using T4 DNA Ligase. The resulting ligation products were transformed into *E. coli* Top10 competent cells, and the transformed bacteria were screened through PCR and sequencing to verify the successful insertion of the *JmSOC1* promoter into the pHIS2 vector. The correctly constructed recombinant vector, now containing the *JmSOC1* promoter integrated into the pHIS2 backbone, was designated as the bait vector for yeast one-hybrid assays and named pHIS2-SOC1.

The interaction assay involving the prey vector pGADT7-SVP and the bait vector pHIS2-SOC1, along with the self-activation assay (pGADT7-Rec2 and pHIS2-SOC1), positive control (pGADT7-Rec2-p53 and pHIS2-p53), and negative control (pGADT7-Rec2 and pHIS2-p53), were co-transformed into yeast competent cells Y187. Following the transformation, the yeast solutions were plated onto DDO medium (SD/-Leu/-Trp) to select for successful co-transformation events and onto TDO/3-AT medium (SD/-His/-Leu/-Trp). The plates were incubated upside down at 30 °C for 2–4 days to allow for colony growth.

## 5. Conclusions

In this study, we cloned the coding DNA sequence (CDS) of the *JmSOC1* gene, a crucial regulator of flower bud development in *J. mandshurica*, which consisted of a 705 base pairs (bp) sequence that encodes a protein of 234 amino acids. Expression of the *JmSOC1* gene was the highest in flower buds. The peak expression level of *JmSOC1* during the physiological differentiation phase occurred earlier in MPD on 10th April compared to FPD on 14th April; similarly, the peak expression in FPG on 2nd April preceded that in MPG on April 14th. This may be the primary reason for the earlier differentiation of the male flowers in protandry individuals and the female flowers in protogyny individuals. Overexpression of *JmSOC1* in wild-type *A*. *thaliana* resulted in earlier flowering, accompanied by an upregulation of key flowering-related genes such as *LFY*, *AP1* and *FT*. To further explore the function of *JmSOC1*, a 782 bp promoter sequence of *JmSOC1* gene was cloned, and the interaction between the *JmSOC1* gene promoter and its upstream regulatory protein JmSVP was verified using yeast one-hybrid. These findings provide valuable insights into the molecular mechanisms promoting early flowering in *J. mandshurica* and offer promise for establishing a theoretical foundation for the flowering regulation network of this species.

## Figures and Tables

**Table 1:** Phenotypic statistics of *Arabidopsis thaliana* plantlets from different sources.

Plant Sources	Bolting Time/d	Flowering Time/d	Number of Rosette Leaves	Number of Lateral Branch	Diameter of Bolting/cm	Plant Height/cm
WT	15.07 ± 0.73 b	36.80 ± 0.64 c	11 ± 1.00 c	5.33 ± 1.53 bc	0.53 ± 0.12 c	23.88 ± 1.70 c
*soc1*	20.79 ± 0.62 a	50.62 ± 0.98 a	19 ± 2.00 a	10.67 ± 2.00 a	0.47 ± 0.03 c	29.38 ± 0.93 a
OE12	10.67 ± 1.34 c	29.05 ± 0.30 d	7.67 ± 1.53 d	4.33 ± 0.57 c	0.89 ± 0.26 a	26.56 ± 0.58 b
C9	16.35 ± 2.79 b	40.98 ± 0.50 b	14.33 ± 1.53 b	7.67 ± 1.53 b	0.67 ± 0.12 b	25.37 ± 0.59 b

WT, wild-type; *soc1*, *soc1* mutant; OE, overexpressed *JmSOC1* plants; C, *soc1* mutant complemented with the *JmSOC1.* The values represent mean ± sd, lowercase letters in the column represent significant difference at α = 0.05 among the different sources of the plantlets.

## Data Availability

Data supporting the findings of this study are available from the corresponding author upon reasonable.

## Supplementary Materials

The following supporting information can be downloaded at: https://www.mdpi.com/article/10.3390/ijms252312932/s1.
