# Supplementary material for: Gene and Its Promoter Cloning, and Functional Validation of JmSOC1 Revealed Its Role in Promoting Early Flowering and the Interaction with the JmSVP Protein
Source: Int J Mol Sci. 2024 Dec 1;25(23):12932. doi: 10.3390/ijms252312932 (PMC11641131; doi:10.3390/ijms252312932)
Supplement: Supplementary file 1 [file ijms-25-12932-s001.zip › Revised-Supplementary TableS1-S2, and Figure legends of Figure S1-S2.pdf]

Figure S1 Amino acid sequence alignment of JmSOC1 and its homologous sequences in other species. The MADS-box domain, K-domain, and SOC1 motif are marked with solid lines, double solid lines, and rectangle, respectively. Potential nuclear localization signals within the JmSOC1 amino acid sequence are marked with dotted lines.

Figure S2 Partial sequence alignment of the *SOC1* gene promoter of *Juglans mandshurica* and *Juglans regia*.

Figure S3 Verification of homozygosity for *soc1* mutants of *Arabidopsis thaliana* (Columbia ecotype) were conducted using the triple primer method (A) and phenotypic observation (B).

Figure S4 Screening of transgenic *Arabidopsis thaliana* resistance using Kanamycin (Kan). A: T1 generation plants. B: T2 generation plants

Figure S5 PCR identification of transgenic plantlets. M, D2000 Marker. -, negative control; +, positive control; o, Blank control; A, T1 generation; B, T2 generation; C, T3 generation.

Table S1 The CDS of *JmSOC1* genes and amino acid sequences of JmSOC1

Name	Sequence
CDS for *JmSOC1*	*ATGTGTGTTTGCTGTCATAG*TGAGTCTCATTTTCTTCTCAAAGGTGT<br>AGAGATGGTGAGAGGAAAGACTCAGATGAGGCGCATAGAGAAC<br>GCCACAAGCAGGCAAGTGACCTTCTCCAAGAGGCGAAATGGGCT<br>GCTAAAGAAAGCGTTTGAGCTATCAGTGCTGTGTGATGCCGAGG<br>TTGCACTCATAATTTTCTCTCCCAGGGGCAAGCTCTATGAATTTG<br>CAAGTTCCAGCATGCAGGAGACAATAGATCGCTACCGGAGGCAC<br>ACAAAAGAGATTCAAATTAACAATAAATCTGTTGAACAAAACAT<br>GCAGCACTTGAGGATTGAATCAACAAACATGATGAAGAAGATTG<br>AGCTTCTTGAAGTTTCGAAACGGAGACTCCTGGGAGAAGGCTTG<br>GGATCATGCTCTATTGAAGAGCTACAACAGATAGAACAACAGTT<br>AGAGAGGAGCGTAAACAAAGTTAGAGCAAGAAAGACTCAGGTT<br>TTCAAGGAACAAATCGAGCAACTAAAAGAGAAGGAGAAAGCTC<br>TAGCAGAGGAAAACGCAAGGCTATCCGAGAAGTGTGGTATTCAA<br>TTACCACAGACAGCAATAAATGAGAATAGAGAAATTTCTGCTTA<br>CGAAGAAAGTACTCCGAGTTCAGATGTGGAGACTGAATTGTTCA<br>TTGGACTGCCGGAAAGGAGAGCAAAGCGCCTCCCACAGAATTGA
Amino acid<br>for JmSOC1	MCVCCHSESHFLLKGVEMVRGKTQMRRIENATSRQVTFSKRRNGL<br>LKKAFELSVLCDAEVALIIFSPRGKLYEFASSSMQETIDRYRRHTKEI<br>QINNKSVEQNMQHLRIESTNMMKKIELLEVSKRRLLGEGLGSCSIEE<br>LQQIEQQLERSVNKVRARKTQVFKEQIEQLKEKEKALAEENARLSE<br>KCGIQLPQTAINENREISAYEESTPSSDVETELFIGLPERRAKRLPQN

Table S2 Distribution of cis-acting elements in the promoter of *JmSOC1* genes

Site	Motif sequence	Function	Quantity
ARE	AAACCA	anaerobic inducible regulatory element	1
Box 4	ATTAAT	partial DNA conserved light response element	1
CAAT-box	CAAT/ CAAAT	promoter and enhancer regions are common cis-acting elements	7
CArG-box motif	CAATATATAG /CCTTTATAGG	the binding site of flowering associated proteins	2
G-Box	TCCACATGGCA /CACGTC	photoresponsive cis-regulating element	1
GATA-motif	GATAGGA /AAGGATAAGG	part of an optical response element	6
GT1-motif	GGTTAA/ GGTTAAT	light responsive element	1
GTGANTG10	GTGA	late pollen gene promoter	1
LTR	CCGAAA	low temperature responsive cis-acting element	1
MBS	CAACTG	MYB binding sites under drought induction	1
MRE	AACCTAA	MYB binding sites associated with light response	1
MYB	CAACAG	associated with drought, salt, and abscisic acid responses	1
MYC	CATGTG	associated with drought and abscisic acid response	1
POLLEN1LELAT52	AGAAA	cis-acting elements of pollen specific expression	6
P-box	CCTTTTG	gibberellin-responsive element	1
TATA-box	TATA/ TATAA/ ATTATA/ATAAAA	transcription start site-30 core promoter elements	22
TATC-box	TATCCCA	cis-acting element involved in gibberellin-responsiveness	1
WRE3	CCACCT	damage response element	2

Table S3 Primers sequences for cloning and functional analysis of *JmSOC1* and its promoter of *Juglans mandshurica*

primers	Sequences (5′-3′)	usage
SOC1-F1	ATGTGTGTTTGCTGTCATAG	*JmSOC1* cloning
SOC1-R1	TCAATTCTGTGGGAGGCGCT	
SOC1-F2	GCTCTAGAATGTGTGTTTGCTGTCATAG	Subcellular localization
SOC1-R2	TCCCCCGGGATTCTGTGGGAGGCGCTTTG	
SOC1-F3	GCTCTAGAATGTGTGTTTGCTGTCATAG	*JmSOC1* expression vector construction
SOC1-R3	TCCCCCGGGTCAATTCTGTGGGAGGCGCT	
SOC1-P-F1	TTTCCGTTTGCCATCC	*JmSOC1* promoter cloning
SOC1-P-R1	AGGTCACTTGCCTGCT	
SOC1-P-F2	CCCAAGCCTTTTCCGTTTGCCATCC	*JmSOC1* promoter vector construction
SOC1-P-R2	TCCCCCGGGAGGTCACTTGCCTGCT	
DSOC1-F	AAACGCAAGGCTATCCGAGAA	qRT-PCR
DSOC1-R	CCGGCAGTCCAATGAACAATT	
Actin-F	ATGCCCAGAAGTCTTGTTCC	
Actin-R	TGCTCATACGGTCAGCGATA	
AtAP1-F	GCTCTTAAGGCACATCCGCAC	
AtAP1-R	GCAGAGGGGGAGGCATATTG	
AtFT-F	ATGCCCAGAAGTCTTGTTCC	
AtFT-R	TGCTCATACGGTCAGCGATA	
AtLFY-F	GACGCAGGTCAAGGAAAGATG	
AtLFY-R	CCAGGCTCCGTTACGATAAA	
AtSVP-F	GAACAAGCGGTTGAGGCA	
AtSVP-R	AGTTTCCGGCGTTAGTAATAGA	
ADSVP-F	GAGTGGCCATTATGGCCCATGGCGAGGGAGAAGATAAA	pGADT7-Rec2-JmSVP prey vector construction
ADSVP-R	GCCGACATGTTTTTTCCCTCAGGTAGAGAACGGAAGCC	
HISpSOC1-F	TCCCCCGGG TTTCCGTTTGCCATCC	pHIS2-pSOC1 bait vector construction
HISpSOC1-R	CGAGCTC AGGTCACTTGCCTGCT	
