# Supplementary figures and images for: Gene and Its Promoter Cloning, and Functional Validation of JmSOC1 Revealed Its Role in Promoting Early Flowering and the Interaction with the JmSVP Protein
Source: Int J Mol Sci. 2024 Dec 1;25(23):12932. doi: 10.3390/ijms252312932 (PMC11641131; doi:10.3390/ijms252312932)

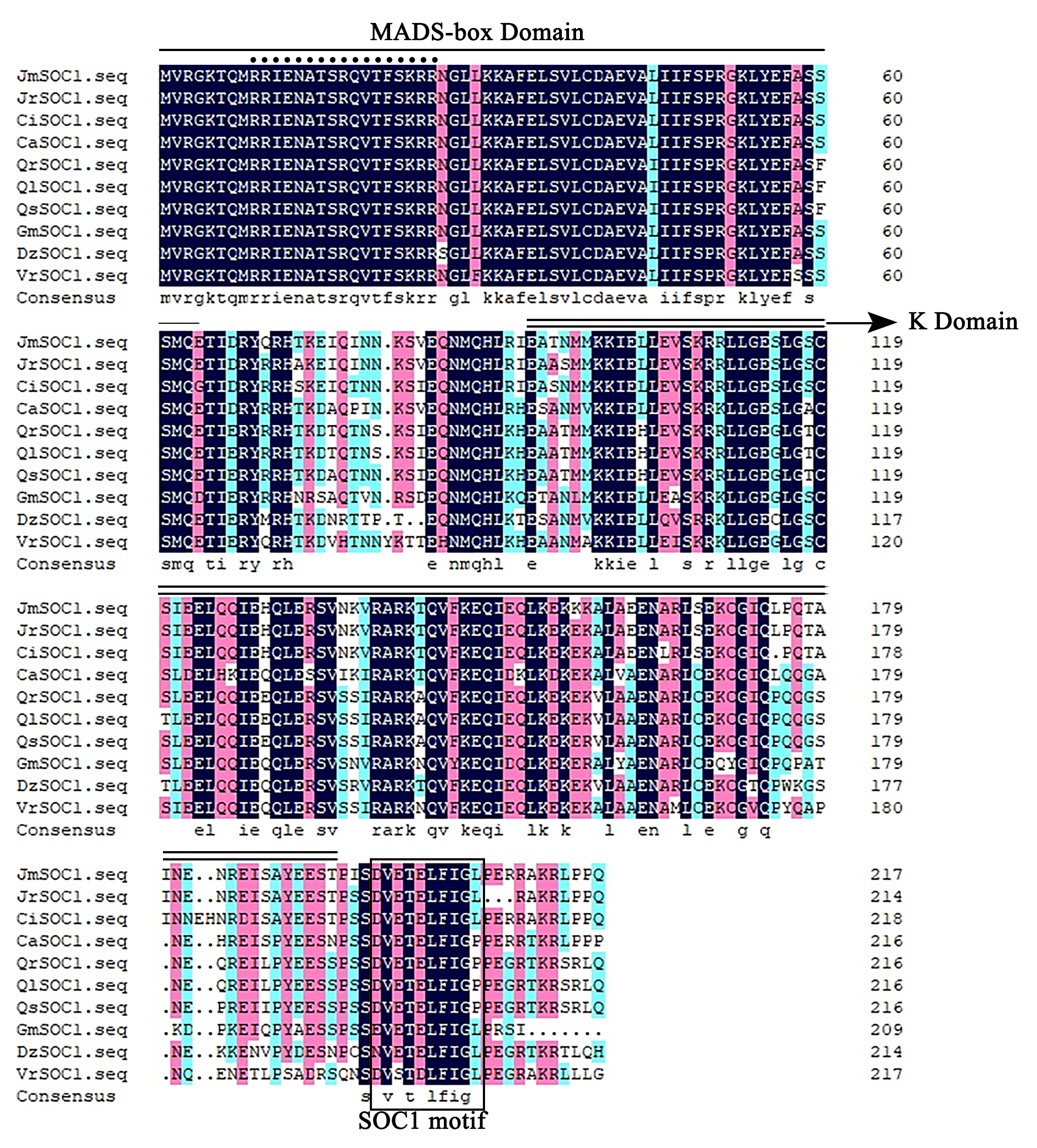

Supplement: Supplementary file 1 [file ijms-25-12932-s001.zip › Figure S1.jpg]

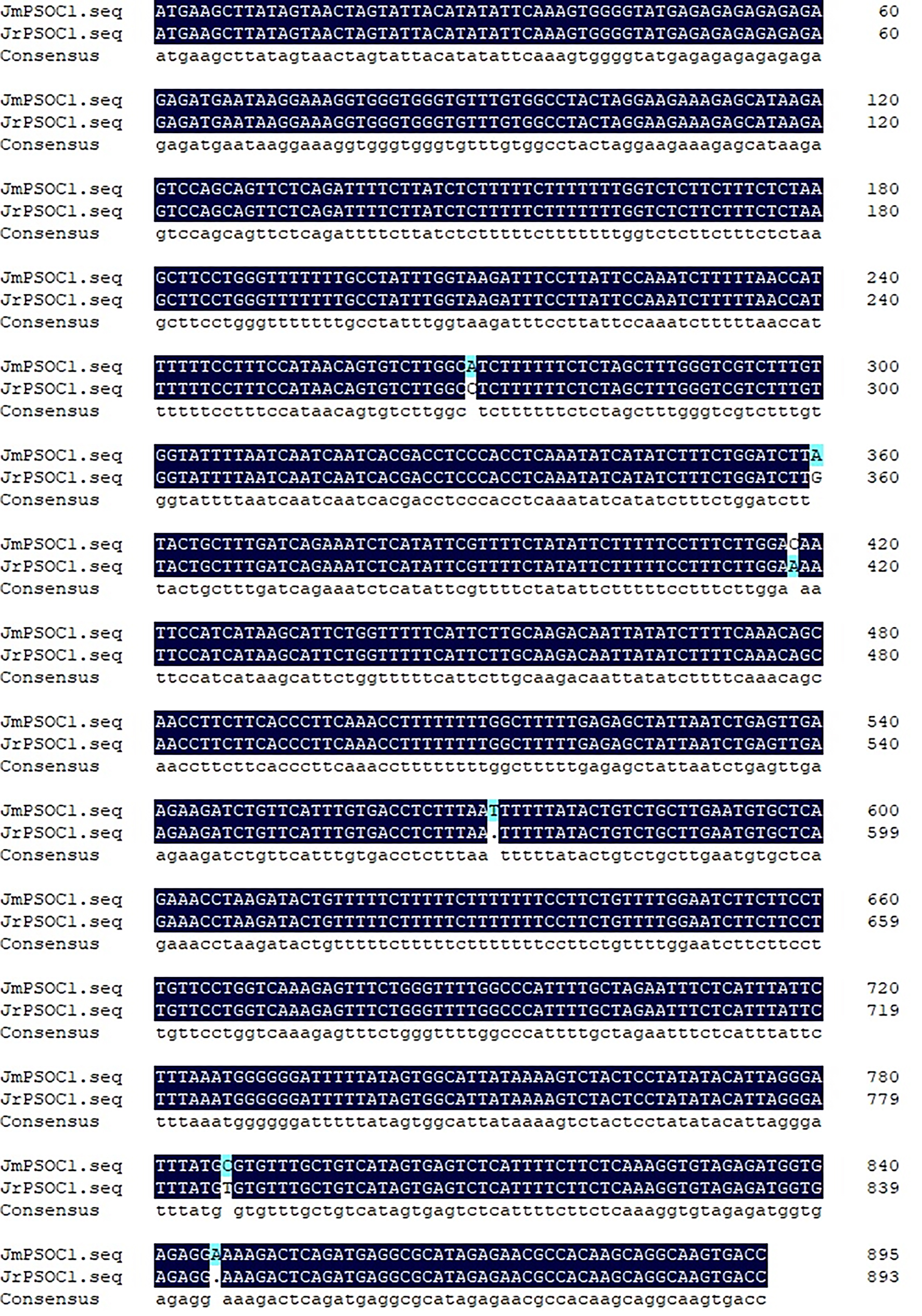

Supplement: Supplementary file 1 [file ijms-25-12932-s001.zip › Figure S2.jpg]

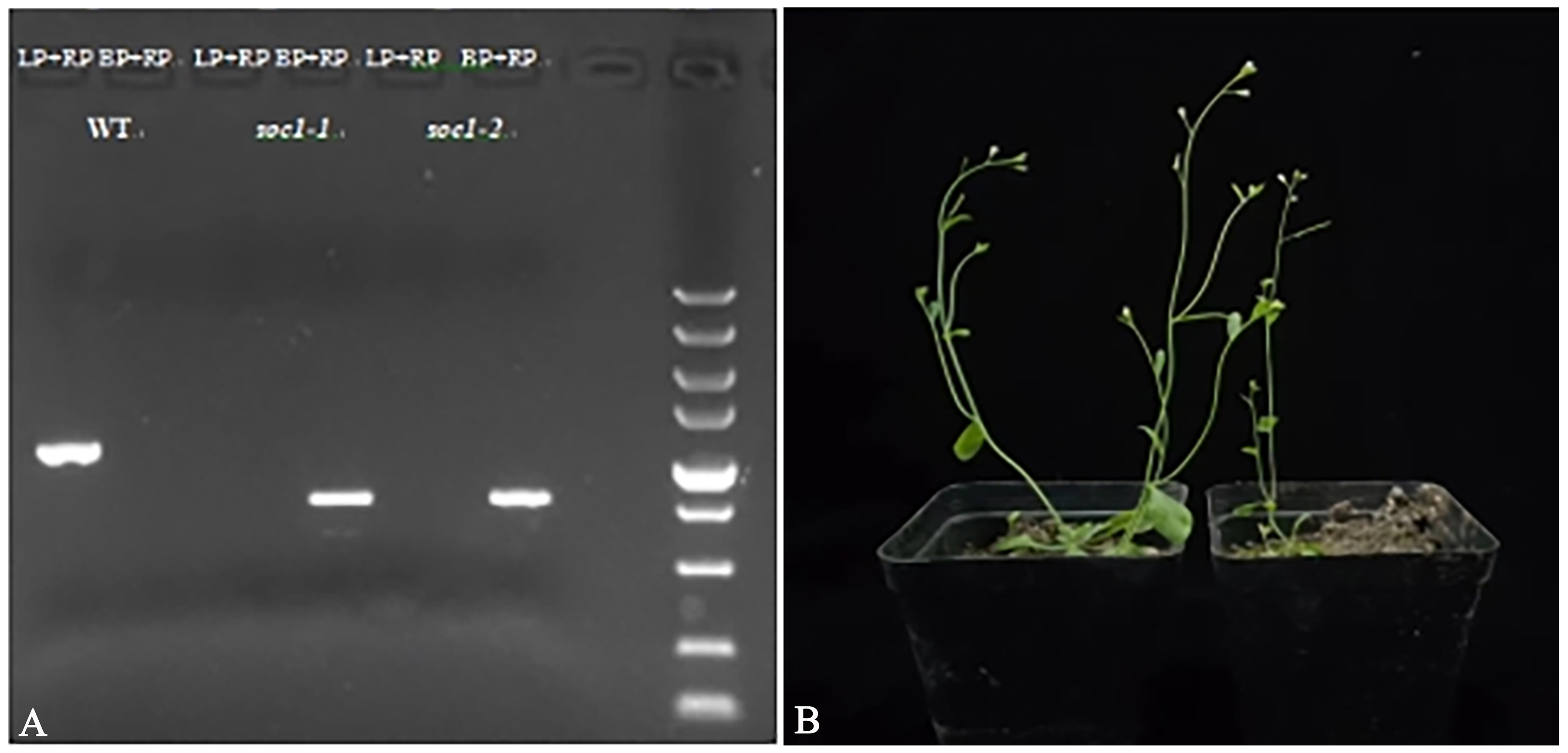

Supplement: Supplementary file 1 [file ijms-25-12932-s001.zip › Figure S3.jpg]

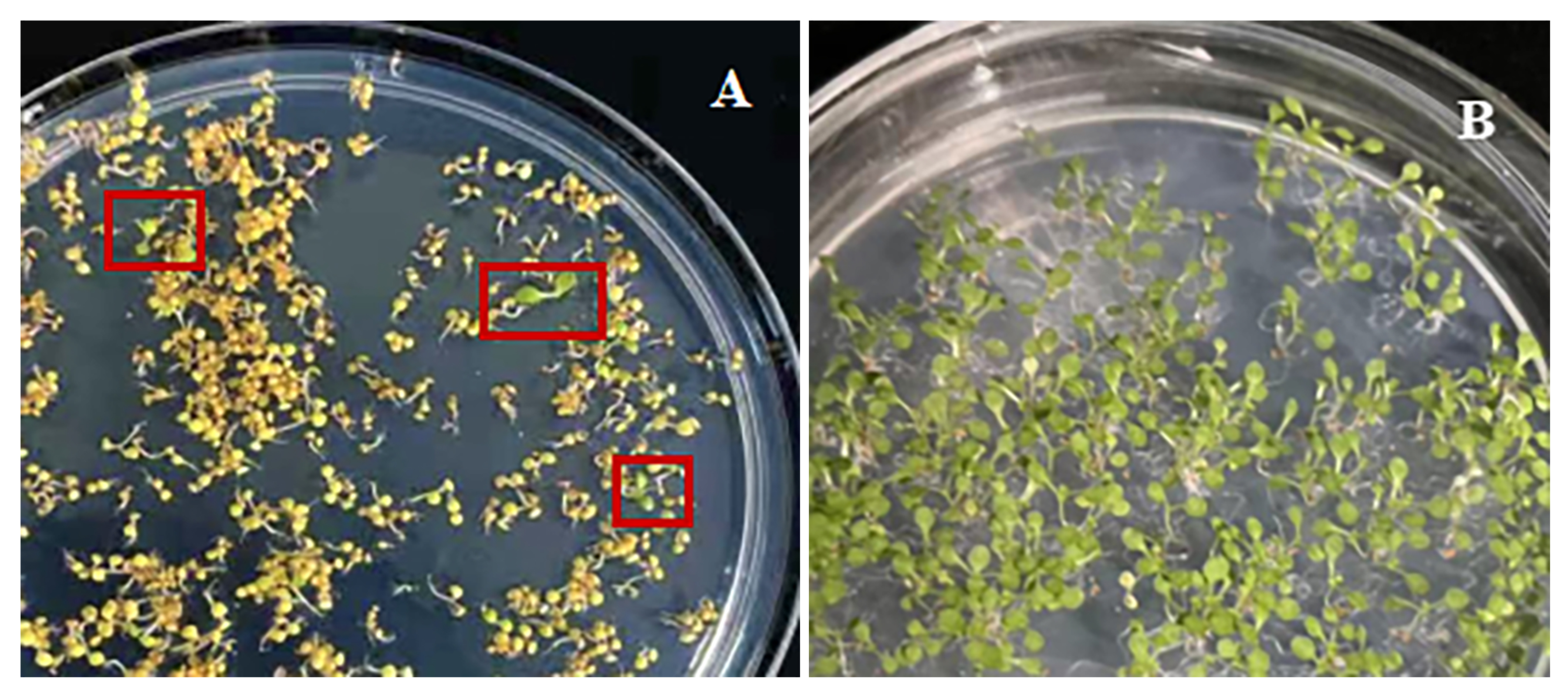

Supplement: Supplementary file 1 [file ijms-25-12932-s001.zip › Figure S4.jpg]

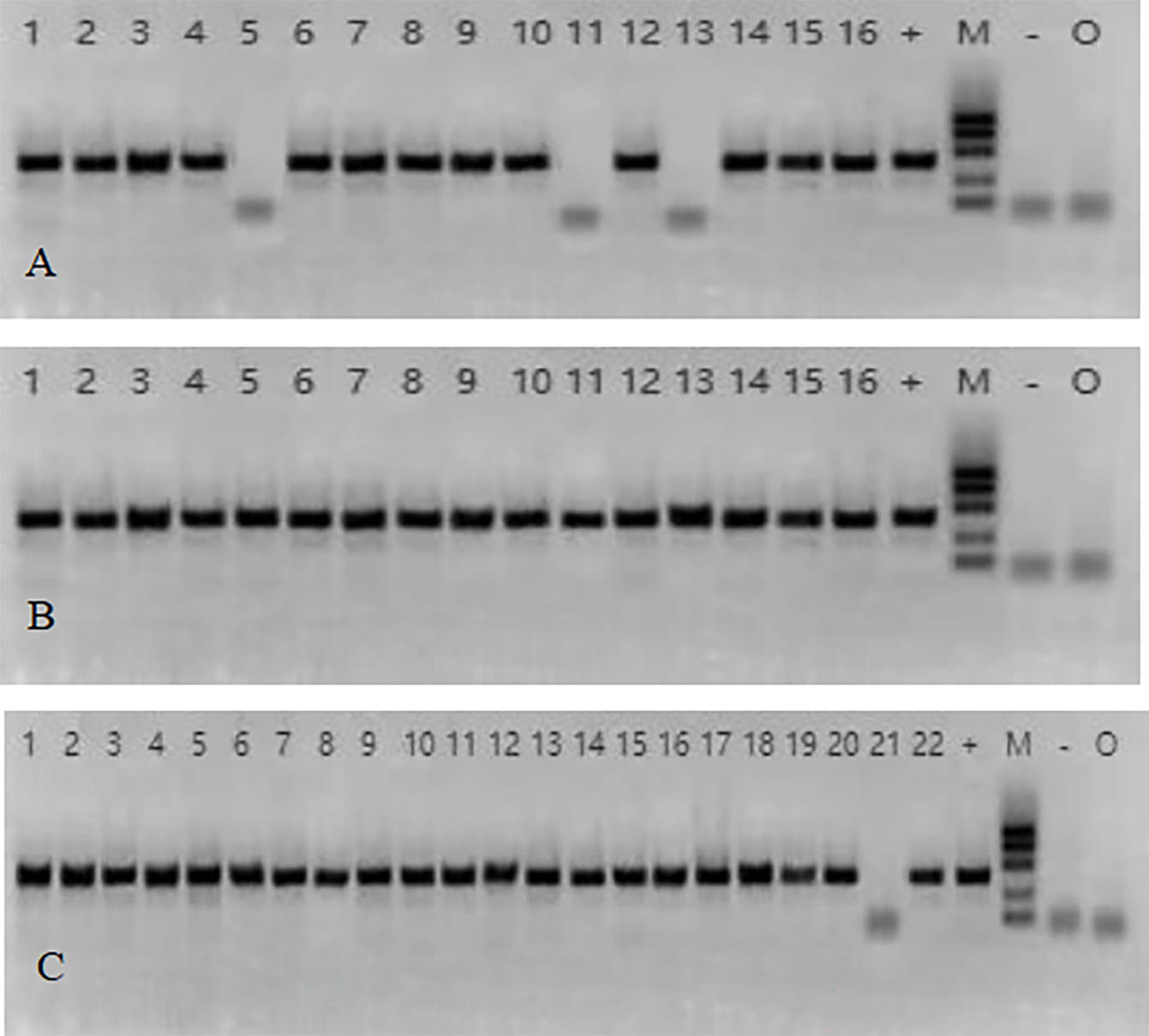

Supplement: Supplementary file 1 [file ijms-25-12932-s001.zip › Figure S5.jpg]
